# Control of *Listeria monocytogenes* in chicken dry-fermented sausages with bioprotective starter culture and high-pressure processing

**DOI:** 10.3389/fmicb.2022.983265

**Published:** 2022-09-30

**Authors:** Anna Austrich-Comas, Cristina Serra-Castelló, Anna Jofré, Pere Gou, Sara Bover-Cid

**Affiliations:** ^1^Food Safety and Functionality Program, Institute of Agrifood Research and Technology (IRTA), Monells, Spain; ^2^Food Quality and Technology Program, Institute of Agrifood Research and Technology (IRTA), Monells, Spain

**Keywords:** *Latilactobacillus sakei* CTC494, biopreservation, corrective storage, innovative food, predictive microbiology, ready-to-eat food, challenge test

## Abstract

*Listeria monocytogenes* is one of the most relevant pathogens for ready-to-eat food, being a challenge for the food industry to comply with microbiological criteria. The aim of the work was to assess the behavior of *L*. *monocytogenes* in two types of chicken-based dry-fermented sausages during the fermentation and ripening, with or without a bioprotective starter culture (*Latilactobacillus sakei* CTC494). To complement the challenge testing approach, simulations with different predictive models were performed to better understand the role of contributing factors. The impact of post-processing strategies, such as high-pressure processing and/or corrective storage was assessed. The chicken meat was inoculated with a cocktail of three *L. monocytogenes* strains, mixed with other ingredients/additives and stuffed into small (snack-type) or medium (*fuet*-type) casings. Snack-type was fermented (22°C/3 days) and ripened (14°C/7 days), while *fuet*-type was ripened (13°C/16 days). At the end of ripening, HPP (600 MPa/5 min) and/or corrective storage (4 or 15°C/7 days) were applied. The suitability of HPP after fermentation was evaluated in the snack-type sausages. Pathogen growth (>3 Log_10_) was observed only during the fermentation of the snack type without a starter. The bioprotective starter prevented the growth of *L. monocytogenes* in the snack-type sausages and enhanced the inactivation (1.55 Log_10_) in *fuet*-type sausages, which could be related to the higher lactic acid production and consequent decrease of pH, but also the production of the antilisterial bacteriocin sakacin k. The gamma concept model allowed us to identify the main factors controlling the *L. monocytogenes’* growth, i.e., the temperature during the early stages and *a*_*w*_ at the end of the production process. The earlier acidification linked with the addition of starter culture made the interaction with the other factors (undissociated lactic acid, *a*_*w*_ and temperature) to be the growth-preventing determinants. High-pressure processing only caused a significant reduction of *L. monocytogenes* in snack-type, which showed higher *a*_*w*_. The application of HPP after fermentation did not offer a relevant advantage in terms of efficacy. Corrective storage did not promote further pathogen inactivation. The findings of the work will guide the food industry to apply effective strategies (e.g., fermentation temperature and bioprotective starter cultures) to control *L. monocytogenes* in chicken dry-fermented sausages.

## Introduction

Dry-fermented sausages (DFSs) are traditional foods highly appreciated by consumers and are considered shelf-stable products as a result of the hurdle technology (Leistner, 2000). As a ready-to-eat (RTE) food, their microbiological safety relies mainly on the control of the pathogens, from raw materials to post-processing, to inhibit their growth and also to promote their inactivation (Serra-Castelló et al., 2021). The traditional processing of DFS evolves under industrial settings linked to the market demands, costs and consumer preferences. For instance, due to lower fat content, the substitution of beef and pork (meat and fat) with skinless poultry meat has been proposed. Moreover, compared with other types of meat (e.g., beef), poultry has lower costs and fewer restrictions due to cultural and religious reasons (Menegas et al., 2013). The ubiquity, persistence, and resistance of one of the main foodborne pathogens, *Listeria monocytogenes*, throughout the manufacturing process of DFS (Martin et al., 2011; Roccato et al., 2017), constitutes a concern for the food industry as well as for the food safety authorities. Some studies reported a prevalence of *L. monocytogenes* in the final DFS product between 5 and 20% (Lebert et al., 2007; Cabedo et al., 2008; Martin et al., 2011; Meloni, 2015), being the cause of a high number of food safety alerts of the Rapid Alert System for Food and Feed (RASFF, i.e., 2021.5188, 2021.6688). Within the food safety management systems, the safety of modified formulations and process conditions need to be carefully assessed, validated and periodically verified (CAC, 2008). As final products, any innovative RTE DFS should comply with the microbiological food safety criteria related to *L. monocytogenes*. In the EU (European Commission, 2005), Canada (Health Canada, 2011), Chile (Ministerio de Salud, 2019), and Australia (FSANZ, 2018), *L. monocytogenes* levels must not exceed 100 cfu/g in DFS along their shelf-life, provided that they do not support the growth of the pathogen. According to the EU regulation, DFS with pH ≤ 4.4 or aw ≤ 0.92 or DFS with pH ≤ 5.0 and aw ≤ 0.94 are automatically considered to belong to food category 1.3. of RTE food unable to support the growth of *L. monocytogenes*, other than those intended for infants and for special medical purposes (European Commission, 2005). This criterion is in line with the Codex Alimentarius standard for *L. monocytogenes* (CAC, 2007), as well as the ISO standard for fermented meat products (ISO, 2021). More restrictive criteria, based on zero tolerance, are required by the US regulation, as *L. monocytogenes* must not be detected in RTE meat products released in the market (FSIS, 2012).

The prevalence and persistence of *L. monocytogenes* in the poultry chain have been reported, being the contamination more frequently related to slaughtering and processing steps (Aury et al., 2011; Iannetti et al., 2020). Different technological strategies to control *L. monocytogenes* during the production of DFS can be applied by food business operators. Biopreservation strategy, understood as the use of microorganisms and/or their metabolites to extend shelf-life and improve food safety, is an inherent trait of food fermentation, which can be enhanced using starter cultures as ingredients of DFS (Barcenilla et al., 2022). Indeed, besides the unspecific mechanisms of microbial interaction, some strains produce specific compounds (i.e., bacteriocins) with bioprotective action against *L. monocytogenes* (Rubio et al., 2013). For instance, while a pediocin and bavaricin (produced by *Pediococcus acidilactici* and *Latilactobacillus curvatus* strains, respectively, included in the commercial Bactoferm F-LC preparation, Chr. Hansen) showed a listeriostatic effect during chorizo fermentation, the sakacin K produced by *Latilactobacillus sakei* CTC494 strain had a listericidal effect against *L. monocytogenes* reducing up to 2 Log_10_ units the pathogen concentration (Ortiz et al., 2014). However, the efficacy of a bioprotective culture depends on the production process conditions as well as the food matrix properties, which makes it necessary to do case-by-case assessments (Barcenilla et al., 2022).

A strategy to control *L. monocytogenes* at the end-product, before market release, is the corrective storage, which takes advantage of the metabolic exhaustion of pathogens exposed to growth limiting *a*_*w*_ (water activity) values at room temperature. For instance, Serra-Castelló et al. (2020) found out that 6 days at 25°C would reduce 1 Log_10_ levels of *L. monocytogenes* in Iberian dry-cured ham (*a*_*w*_ < 0.87). Another lethality strategy to increase the food safety of RTE meat products is the application of non-thermal high-pressure processing (HPP) (Buchanan et al., 2017; Bover-Cid et al., 2019). However, the efficacy of HPP depends on the product’s characteristics, for example, low *a*_*w*_ is known to reduce the lethal effect of HPP (Bover-Cid et al., 2015; EFSA BIOHAZ Panel, Koutsoumanis et al., 2022). Therefore, it is necessary to assess the impact of these technological strategies through a product-oriented approach, taking into consideration the processing conditions and the characteristics of the final product.

Challenge testing is a methodology widely used to investigate the behavior of pathogens inoculated in a food matrix that is submitted to study conditions mimicking the industrial process (Bonilauri et al., 2019; Bover-Cid et al., 2019; Serra-Castelló et al., 2020). Predictive models available in the scientific literature could also be used to simulate the behavior of pathogens during the production process and their subsequent storage period introducing the physico-chemical characteristics of the product as inputs (Walls and Scott, 1997; Fakruddin et al., 2012; Pérez-Rodríguez and Valero, 2013; Guillier, 2016; Bover-Cid et al., 2019).

In this framework, the aim of the study was to assess the behavior of *L. monocytogenes* during the production of two types of low acid chicken DFS of different caliber (a *fuet* type and a snack type), with or without the use of starter culture consisting of a bioprotective antilisterial strain (*Latilactobacillus sakei* CTC494). The results of the challenge tests were compared with the predictions provided by predictive models currently available in the literature to simulate the behavior of *L. monocytogenes* in DFS. In addition, the effect of a subsequent corrective storage period and/or the application of HPP at different process times were explored.

## Materials and methods

### Bacterial strains

Strains of *L. monocytogenes* used in the present study included: a dry-cured meat product isolate CTC1034 (serotype 4b) from the IRTA culture collection used in previous studies (Bover-Cid et al., 2011, 2019; Hereu et al., 2012; Hereu et al., 2014); Scott A (4b): a clinical strain frequently included in challenge tests dealing with meat products (Van Boeijen et al., 2010; Bover-Cid et al., 2019); and 12MOB045LM (genoserotype II): selected from the set of characterized strains from the European Reference Laboratory for *L. monocytogenes* (EURL-Lm, Guillier et al., 2013). The strains CTC1034 and Scott A belong to clonal complex CC1 and CC2, respectively (Martin et al., 2014), while strain 12 MOB045LM belongs to CC9 [reported with strain ID 14SEL860LM in Fritsch et al. (2019), personal communication by L. Guillier]. Each strain was independently cultured in Brain Heart Infusion (BHI) broth (Becton Dickinson, Sparks, MD, USA) at 37°C for 24 h and preserved at −80°C with 20% glycerol as cryoprotectant until being used. Thawed cultures of each strain were used to inoculate the meat batter.

The sakacin K-producing strain *L. sakei* CTC494 (Hugas et al., 1995) was used as a bioprotective starter culture, which was grown up in MRS broth at 30°C for 24 h and preserved at −80°C with 20% glycerol as cryoprotectant until being used.

### Preparation, processing and storage conditions of chicken-based DFS

Minced chicken meat (100%) as raw material for DFS manufacture was obtained directly from the DFS producer and transported to IRTA premises under refrigeration (2°C). No additional fat was used. The meat (26 kg) was inoculated with *L. monocytogenes* (1% v/w) using a cocktail prepared by mixing equal concentrations of each strain (section “Bacterial strains”) diluted in physiological saline (0.85% NaCl and 0.1% Bacto Peptone) to reach a final concentration of *ca*. 6 Log_10_ cfu/g. The inoculated meat batter was homogenized for 1.15 min in a mixing machine (Mix-35P, Tecnotrip, Spain). DFS ingredients provided by the DFS producer were added to the batter according to the innovative recipe, including maltodextrin, glucose syrup, salt, spices, antioxidant (sodium ascorbate), beet concentrate, flavor, preservatives (potassium nitrate and sodium nitrite, each with an in-going amount of 140 mg/kg) and commercial culture of *Staphylococcus xylosus* (Lyocarni SXH-38, Sacco System, Cadorago, Italy) and mixed for an additional 135 seconds. In half of the batter, *L. sakei* CTC494 was added at *ca*. 6 Log_10_ cfu/g and mixed for 90 seconds, while in the other half the same amount of sterile water (without lactic acid bacteria) was added.

The meat batter was stuffed using a stuffing machine (H15, Tecnotrip, Spain) in two different casings to produce two chicken DFS types: a natural pork casing (40–42 mm caliber) was used for *fuet* type (FT), while an edible collagen casing (14 mm caliber) was used for the snack type (ST). Both types of sausages were dipped into a solution of *Penicillium nalgiovense* (Meat Surface PS 521, Lallemand Specialty Cultures, France) according to the manufacturer’s instructions.

**FIGURE 1:**
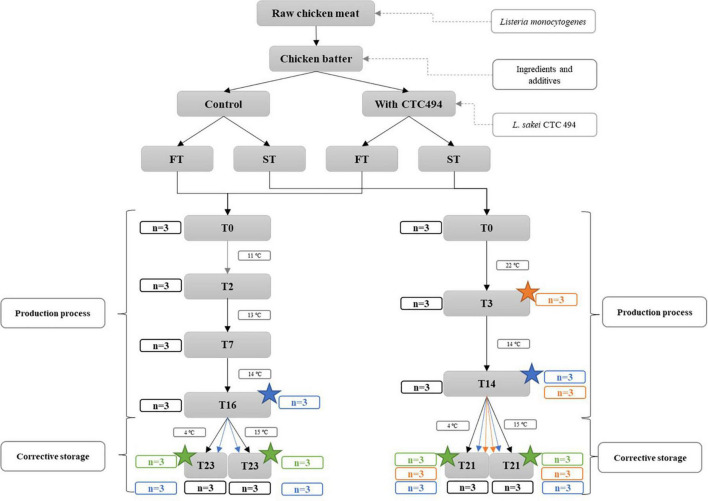
Summary of the manufacturing process and sampling scheme of *fuet*-type (FT) and snack-type (ST) fermented sausages (*n* is the number of sampled sausages at each point). Application of high-pressure processing (HPP) is indicated with a star.

The fermentation and ripening conditions were different for each product type and mimicked the industrial conditions applied by the DFS producer (Figure 1). FT was slightly fermented and gradually ripened for 2 days at 10–12°C and 76–80% RH, 5 days at 12–14°C and 81–86% RH and 10 days at 13–15°C and 64–70% RH; while ST was fermented for 3 days at 21–23°C and 77–80% RH and ripened for 8 days at 13–15°C and 64–70% RH. Afterward, sausages were kept in PA/PE plastic bags (oxygen permeability of 50 cm^3^/m^2^/24 h and a low water vapor permeability of 2.8 g/m^2^/24 h; Sistemvac, Estudi Graf SA, Girona, Spain) sealed (with air) and stored at 4 or 15°C for up to 7 days.

A single batch was produced for each type of sausage, thus the variability between batches could not be addressed. However, as stated in the ISO 20976-1 (ISO, 2019) standard, the use of a single batch is justified when evaluating the impact of a new formulation of the food and/or it represents the worst-case scenario.

### High-pressure processing

High-pressure processing (HPP) was applied to the final product after the ripening time (days 14 and 16 for ST and FT, respectively) and after 7 days of storage at each temperature. Additionally, for a set of ST sausages, HPP treatment was also applied after the fermentation (day 3), once the lactic acid bacteria reached the exponential growth phase. For each HPP cycle, individual sausages were vacuum-packaged (EV-15-2-CD; Tecnotrip, Terrassa, Spain) in PA/PE plastic bags (Sistemvac, Estudi Graf SA). Samples were subjected to HPP at 600 MPa for 5 min at an initial water temperature of 10°C using a Wave6000 (Hiperbaric, Burgos, Spain) equipment. The pressure come-up rate was 177 MPa/min on average and the pressure release was almost immediate.

### Sampling scheme and analytical determinations

Samples of each type of DFS were periodically analyzed according to the sampling scheme detailed in Figure 1, except for pH that was monitored more frequently. Briefly, three replicate sausages from each batch (with and without starter culture *L. sakei* CTC494) were taken on day 0 (just after stuffing). During the production and storage, samplings were performed just before changing the temperature of the process, i.e., on days 2, 7, 16, and 23 for FT and on days 3, 14, and 21 for ST. A total of 138 sausages were sampled.

The pH was measured with a penetration probe (52-32; Crison Instrument SA, Alella, Spain) connected to a portable pH-meter (PH25; Crison Instruments). The total lactic acid (D- and L-lactic acid) was determined using the D-/L-Lactic Acid (D-/L-Lactate) Assay kit from Megazyme International (Wicklow, Ireland) following manufacturer instructions. The product’s *a*_*w*_ was measured with an AquaLab™ instrument (Series 3; Decagon Devices Inc., Pullman, WA, USA).

To monitor the behavior of *L. monocytogenes* and lactic acid bacteria (LAB), 15 g of sausage were 10-fold diluted and homogenized in physiological saline (0.85% NaCl and 0.1% Bacto Peptone) in a bag blender Smasher^®^ (bioMérieux, Marcy-l’Étoile, France) for 60 s. Serial decimal dilutions were prepared in physiological saline. *L. monocytogenes* was enumerated on selective and differential chromogenic agar (CHROMagar™ Listeria, Scharlab, Spain) incubated at 37°C for 48 h. In samples with expected *L. monocytogenes* concentration below the quantification limit (<10 cfu/g), the detection/not detection of *L. monocytogenes* was confirmed after the TSBYE homogenate enrichment (at 37°C for 48 h) by plating in chromogenic agar. LAB was enumerated in Man-Rogosa-Sharpe (MRS) agar plates (Merck, Darmstadt, Germany) incubated at 30°C for 72 h anaerobically using sealed jars with AnaeroGen sachets (Oxoid ltd.).

### Statistical analysis

The differences between the mean values of the analytical results for each sausage type, the effect of the addition of bioprotective culture, high-pressure processing, or the storage were tested by means of a *t*-test. The significance level was established at α = 0.05.

### Simulation of the behavior of *Listeria monocytogenes* using predictive models

Different predictive models available in the scientific literature were used to simulate the behavior of *L. monocytogenes* in chicken-based DFS. Mataragas et al. (2015) approach dealt with the non-thermal inactivation of *L. monocytogenes* in DFS according to temperature through an Arrhenius type model, using two specific equations: one for the fermentation process (which was used for the first 3 days of ST), and another for the ripening, which was used for the second part of the process of ST and all the process of FT). Hwang et al. (2009) model evaluates the non-thermal inactivation of *L. monocytogenes* in two steps based on two different polynomial models: the first one considers the pH at the end of the fermentation as an input factor, while the second one considers pH and *a*_*w*_ at the end of the ripening process. Coroller et al. (2012) model is based on the gamma concept, using biological parameters to simulate the effect of the combination of environmental conditions on the microbial growth/inactivation rate based on pH, *a*_*w*_, temperature and undissociated lactic acid concentration (Coroller, 2006). The gamma parameter provides the quantitative impact of each (input) factor on the inhibition of the pathogen growth. The temperature along the production process and/or the physico-chemical characteristics of the product (pH, *a*_*w*_ and undissociated lactic acid concentration) during the challenge test was introduced as input factors in the mathematical equations of models implemented in MS-Excel.

## Results and discussion

### Physico-chemical characteristics, lactic acid bacteria and *Listeria monocytogenes* counts during sausage fermentation and ripening

**FIGURE 2:**
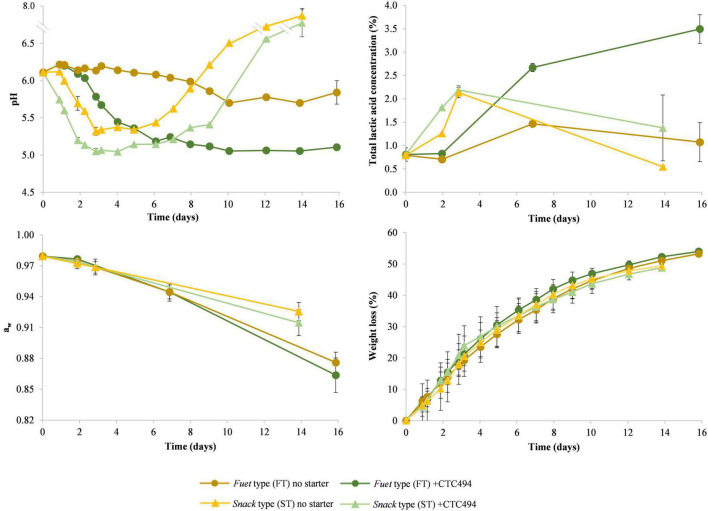
Results of physico-chemical determinations (pH, total lactic acid concentration, *a*_*w*_ and weight loss) during the fermentation and ripening of *fuet*-type (FT) and snack-type (ST) sausages spontaneously fermented (no starter) and with the addition of the bioprotective (sakacin K-producer) *L. sakei* CTC494. Error bars correspond to standard deviation of the mean value.

During the production process, the acidification and drying profile was dependent on the sausage type (which followed specific process conditions) and the use of starter culture. Figure 2 shows the change in pH, total lactic acid concentration, *a*_*w*_ and weight loss during the fermentation and ripening of FT and ST sausages.

In FT without bioprotective starter culture, the pH changed very little, and only a slight gradual decrease (*p* < 0.05) from 6.11 to 5.84 at the end of the ripening was observed, which is related to a relatively little amount of total lactic acid accumulated (1.26%). The low acidity observed in *fuet* corresponds to a worst-case scenario typical for this type of Catalan DFS usually made of pork, which is only slightly fermented along with the ripening at temperatures below 15°C (Rubio et al., 2014). The addition of the bioprotective starter culture (*L. sakei* CTC494) resulted in a drop of the pH from 6.11 to 5.2 between 2 and 6 days, concurring with the accumulation of total lactic acid up to 2.67%, and afterward, the production of lactic acid slowed down and the pH kept around 5.11 till the end of the process. The product’s *a*_*w*_ declined gradually from 0.979 to 0.870 at the end of ripening without significant differences between sausages with and without bioprotective culture (*p* < 0.05), in agreement with the similar weight loss due to the drying process.

In ST sausages, the higher fermentation temperature compared with FT caused an earlier and more intense drop in the pH during the first 3 days, reaching pH 5.32 and 5.05 in sausages without and with the bioprotective starter. At the fermentation temperature applied for ST, a higher amount of total lactic acid was produced, reaching *ca*. 2.16% after 3 days. During the last week, a considerable increase in the pH above 7 was observed, which can be attributed to the decrease of lactic acid concentration and particularly to a thick mold layer covering the surface of the sausages. Though the increase of the pH is typical by the end of the ripening of Mediterranean DFS due to the proteolysis and ammonia production from amino acid (Thévenot et al., 2005; Rubio et al., 2014; Porto-Fett et al., 2022), the magnitude of observed in the present study was not expected. In this type of sausages, the *a*_*w*_ decreased similarly to FT sausages during the first week, however, during the second week, the thick mold layer grown on the surface resulted in a slower drying process (Toldrá et al., 2014), thus resulting in a final product with higher *a*_*w*_ (*p* < 0.05), corresponding to worst-case scenarios for these types of fermented sausages.

**FIGURE 3:**
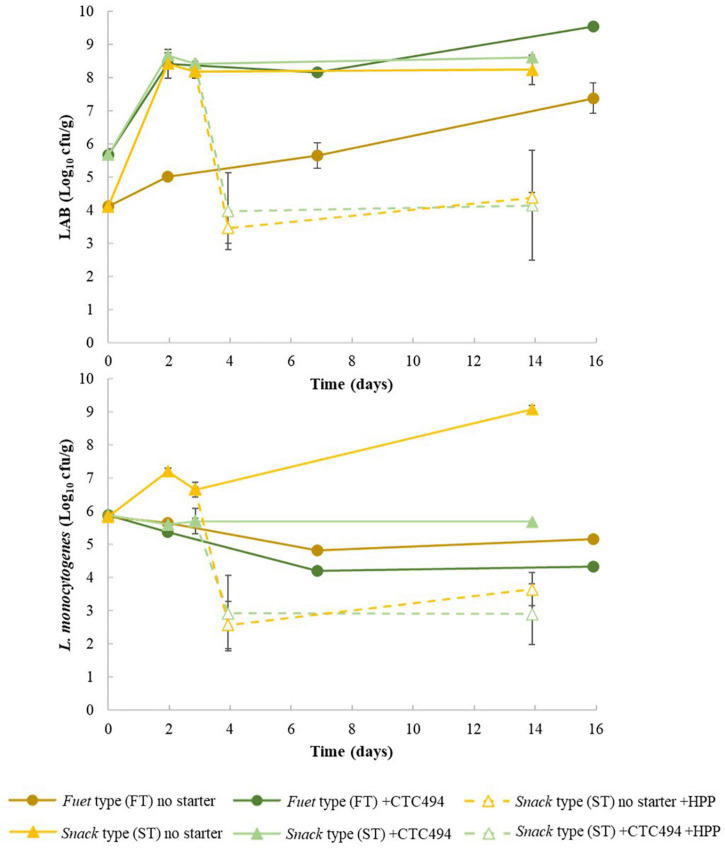
Counts of lactic acid bacteria (LAB) and *L. monocytogenes* during the fermentation and ripening of *fuet*-type (FT) and snack-type (ST) sausages spontaneously fermented (no starter) and with the addition of the bioprotective (sakacin K)-producing *L. sakei* CTC494. Error bars correspond to standard deviation of the mean value.

Lactic acid bacteria levels during the production process for FT and ST sausages are shown in Figure 3. In FT spontaneously fermented, endogenous LAB population were at 4 Log_10_ cfu/g and grew slowly, e.g., only 0.89 Log_10_ after 2 days, reaching 7.4 Log_10_ cfu/g at the end of the process. In FT with bioprotective *L. sakei* CTC494, counts of LAB sharply increased by 2.7 Log_10_ during the first 2 days of the process, reaching and maintaining the maximum population density higher than 8 Log_10_ cfu/g. It is known that *L. sakei* is a psychrotrophic bacteria well adapted to meat fermentation environment, particularly at moderate-low temperatures (10–15°C) applied for the fermentation-ripening of *fuet*-type sausages (Zagorec and Champomier-Vergès, 2017), which explains the rapid and high increase of LAB population in products with the bioprotective strain.

The fate of LAB was affected by the different conditions during the production process of ST (fermentation and ripening). LAB population sharply increased within the first 2 days (*p* < 0.05), reaching a maximum population up to 8.7 Log_10_ cfu/g and 8.4 Log_10_ cfu/g in ST with and without starter culture, respectively, which was maintained during the ripening. The greatest and earliest LAB growth observed in ST compared to FT could be attributed to the higher temperature, which promoted the growth of the spontaneous LAB (in ST without starter) and the bioprotective strain. The different LAB growth profile observed between types of fermented sausages (FT vs. ST), with and without the bioprotective strain of *L. sakei* CTC494, explains the different acidification patterns both in terms of total lactic acid production and the consequent drop of pH.

The behavior of *L. monocytogenes* during the manufacture of FT and ST is shown in Figure 3. In FT sausages, the pathogen could not grow. Rather, a gradual loss of viability during the 2 weeks of the fermentation-ripening process was observed. In the spontaneously fermented FT sausages (without starter culture), about 0.7 Log_10_ reduction was observed. The addition of the bioprotective *L. sakei* CTC494 resulted in a significantly (*p* < 0.05) greater reduction of the pathogen load of about 1.6 Log_10_, which could be related to the higher lactic acid production and consequent decrease of pH, but also to the production of the antilisterial bacteriocin sakacin k (Hugas et al., 1995). No information has been found in the literature about the behavior of *L. monocytogenes* in chicken-based DFS, however, inconsistent results have been published for similar types of DFS made of pork. For instance, though no growth of *L. monocytogenes* was reported during the first week of the production of spontaneously fermented pork *fuet* and chorizo sausages ripened at 12°C to a pH around 5.3, a higher pathogen reduction (about 2 Log_10_) occurred at later stages of the process (Marcos et al., 2005). On the contrary, a considerable increase of *L. monocytogenes* (about 2 Log_10_ units) in *fuet* without starter culture was observed during the first week of ripening at 12°C even if the pH decreased to 5.3 (Garriga et al., 2005) or at 15°C without a significant decrease of pH (Jofré et al., 2009). The addition of starter culture (non-bacteriocinogenic) was reported to be critical to control the growth of *L. monocytogenes*, though only limited reduction (<0.5 Log_10_) of the pathogen was achieved (Garriga et al., 2005).

In ST sausages, the fermentation step at 22°C (first 3 days) supported the growth of the pathogen (*ca*. 1.4 Log_10_ increase) in spontaneously fermented samples. Only when the bioprotective *L. sakei* CTC494 was added the pathogen growth was inhibited, and a slight reduction (*p* > 0.05) of *L. monocytogenes* was observed (from 5.84 to 5.60 Log_10_ cfu/g). These results agreed with the findings of Hugas et al. (1995) dealing with the behavior of *L. monocytogenes* in pork chorizo sausage fermented at 20°C/24 h followed by ripening at 7°C/13 days: a considerable growth of the pathogen was recorded in spontaneously fermented samples, while an overall pathogen reduction (ca. 2 Log_10_ units) was achieved when the bioprotective *L. sakei* CTC494 was added in the formulation.

Degenhardt and Sant’Anna (2007) reported different behaviors of *L. monocytogenes* in low-acid Italian DFS (fermented at 22–24°C/48 h and ripened at decreasing temperature from 22 to 12°C up to 28 days) depending on the source of contamination. In natural contaminated products, *L. monocytogenes* was able to grow (1 Log_10_) at the beginning of the process, while in inoculated products, a constant decrease was observed throughout the experiment. The difference was attributed to the different traits between the meat native (thus better adapted) and culture collection strains. Thévenot et al. (2005) also found a strain-dependent inactivation rate of *L. monocytogenes* during the ripening of French DFS, strains isolated (adapted) from sausage or sausage industry environment decreased by 1.5 Log_10_, while clinical isolates (non-adapted) decreased by more than 3 Log_10_ after 35 days of the drying process. Our experiment was performed using a cocktail of *L. monocytogenes* strains that contained dry-cured meat isolates adapted to fermented sausage conditions, thus covering a worst-case scenario.

### Simulation of *Listeria monocytogenes* behavior through predictive models

**FIGURE 4:**
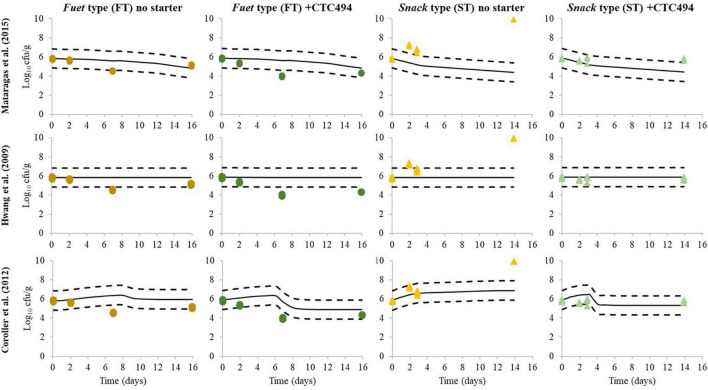
Simulation of *L. monocytogenes* behavior during the fermentation and ripening of *fuet*-type (FT) and snack-type (ST) chicken sausage without starter culture and with the bioprotective *L. sakei* CTC494 culture, applying predictive models available in the scientific literature. Continuous line represents model predictions, dashed line represents ±1 Log_10_ interval and dots represent the experimentally observed results in the present challenge test.

The behavior of *L. monocytogenes* was simulated for each batch according to the three different predictive models available in the literature and the predictions were compared with the experimental results of the challenge test (Figure 4). The Arrhenius-type model proposed by Mataragas et al. (2015) estimates the non-thermal inactivation of *L. monocytogenes* as a function of temperature only. The predictions were reasonably in accordance with the observations in the case of FT, though the model slightly underestimated the effect of the bioprotective culture. However, for ST, the model provides fail-dangerous predictions (inactivation), while growth or no change of *L. monocytogenes* was observed. According to meta-analyses performed by several authors regarding the inactivation rate of bacterial pathogens during sausage fermentation and ripening, the temperature explains most (60–80%) of the variability and, under growth-preventing conditions, determines the reduction of *L. monocytogenes* (Mataragas et al., 2015) and *E. coli* (Ross and Shadbolt, 2001; Ross et al., 2008; Quinto et al., 2014). In these studies, the influence of pH, *a*_*w*_ or other specific factors (e.g., bacteriocins) cannot be discerned from the background noise of all collated data originating from a wide variety of experiments, including different types of products, processes, bacterial strains, etc. Therefore, the temperature-dependent Arrhenius-type model cannot simulate the impact of different pH and *a*_*w*_ profiles occurring in the different types of sausages as a result of the different processing conditions nor the action of the bacteriocin produced by the bioprotective bacteria.

The polynomial models about the Log_10_ reduction of the pathogen proposed by Hwang et al. (2009) consider pH and *a*_*w*_ as input factors, but not the temperature. According to the model predictions, no relevant inactivation of *L. monocytogenes* would occur irrespectively of the different acidification and drying profiles recorded in the challenge test. The mathematical model was developed in and for *soudjouk* type sausage, which is made of beef and with a pH after the fermentation (4.6–5.2) considerably lower than the usual pH of the slightly low acid fermented sausages studied here. Therefore, this model would not be suitable to assess the behavior of *L. monocytogenes* in low-acid Mediterranean-type DFS.

The gamma-approach model followed by Coroller et al. (2012) considers more factors (temperature, *a*_*w*_, pH and undissociated lactic acid). It allows to simulate the potential growth of *L. monocytogenes* at the early stages of the production process and, when the combined conditions do not support growth, the model quantifies the inactivation. Although not observed, a slight increase of 0.44 Log_10_ until day 6 and 0.56 Log_10_ increase until day 8 was predicted for FT with and without starter culture, respectively. Subsequently, the predicted inactivation was greater for FT with starter (1.41 Log_10_ reduction) than without starter (0.42 Log_10_ reduction) due to the higher production of lactic acid and pH decrease when the bioprotective strain was added. Regarding the ST, the predicted growth was quite in agreement with the experimental results during the first step (3 days of fermentation) of sausages without a starter culture. However, the model strongly underestimated the growth of *L. monocytogenes* in the spontaneously fermented ST (0.3 Log_10_ increase predicted compared with about 2 Log_10_ increase observed). In the case of ST with the bioprotective culture, the model predicted a 0.6 Log_10_ increase followed by a reduction of 0.88 Log_10_. These changes are of relatively small magnitude, being comparable with the constant levels of *L. monocytogenes* experimentally observed along the fermentation and ripening.

**FIGURE 5:**
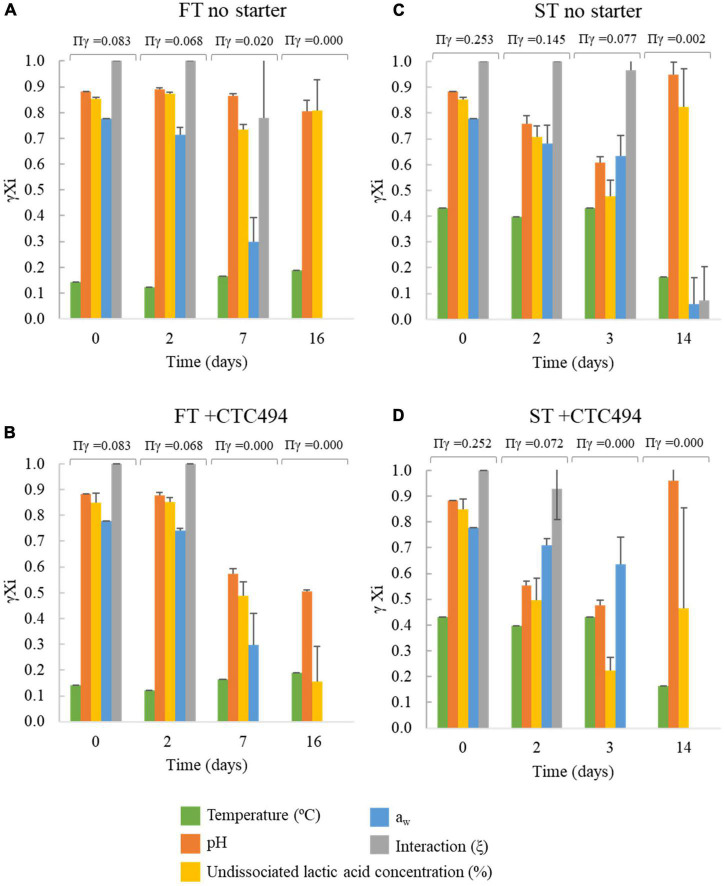
Gamma (γ) values for each environmental factor considered in the predictive model used for simulating *L. monocytogenes* behavior during the fermentation and ripening of *fuet* type (FT) and snack-type (ST) sausages with no starter) **(A,C)** and with the addition of the bioprotective (sakacin K)-producing *L. sakei* CTC494 **(B,D)**. Error bars correspond to the standard deviation of the mean value.

Through the gamma-concept approach, the contribution of each individual factor included in the model and the interaction among factors on the inhibition of *L. monocytogenes* growth can be identified through the gamma (γ) estimates (Figure 5). The lower the γ value, the stronger the inhibitory effect, i.e., when the γ of a given factor or the interaction is zero, the product of the gamma factors (Πγ) is zero meaning that the growth is totally inhibited. A γ = 1 implies that the growth potential is optimal for the given factor. In FT sausages, the temperature of the process (10–12°C) was the main factor limiting the growth of the pathogen during the first days (Figures 5A,B). The contribution of the lowered *a*_*w*_ become relevant on day 7 and was the factor accounting for the growth prevention in the final product (day 16) in the spontaneously fermented sausages (Figure 5A). In FT with a starter culture, the slight acidification resulted in lower γ values for pH and undissociated lactic acid, making the interaction between factors (ξ) fall to 0 (growth prevention) on day 7 (Figure 5B). Regarding the ST sausages, the higher temperature (22°C) during the fermentation was less inhibiting than for FT. At the end of the process in ST spontaneously fermented none of the “γ” values were zero, even the “γ” for aw and the interaction were close to zero, meaning that the growth of *L. monocytogenes* was not totally inhibited (Figure 5C). For ST with a starter culture, the acidification and particularly the amount of undissociated lactic acid showed the lowest γ value accounted for the growth suppression (γ = 0) already on day 3 (Figure 5D).

Under real industrial conditions (using much bigger drying chambers), the products may be exposed to slightly different conditions, which in turn will be subjected to intra-batch as well as batch-to-batch variability. The application of appropriate predictive models would allow assessing the impact of the different pH and *a*_*w*_ decrease resulting from such variability. In this respect, the combination of methodologies when assessing the microbiological safety of food processing conditions (i.e., challenge testing and simulations with predictive models) provides complementary approaches to increase the robustness of outputs (CAC, 2008).

### Impact of post-processing strategies: Corrective storage and/or high-pressure processing

**FIGURE 6:**
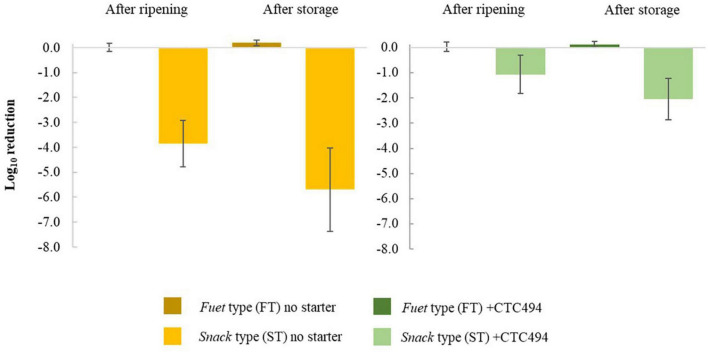
Inactivation (Log_10_ reduction) of *L. monocytogenes* due to the application of high-pressure processing (HPP) after ripening and storage of *fuet*-type (FT) and snack-type (ST) sausages spontaneously fermented (no starter) and with the addition of the bioprotective (sakacin K)-producing *L. sakei* CTC494. Error bars correspond to the standard deviation of the mean value.

The results of the inactivation (expressed as Log_10_ reduction) of the post-processing strategies consisting of corrective storage and/or HPP are shown in Table 1 and Figure 6, respectively.

**TABLE 1:** Inactivation (Log_10_ reduction) of *L. monocytogenes* due to the application of the corrective storage (7 days) at 4 and 15°C after ripening and after a HPP treatment at the end of ripening of *fuet*-type (FT) and snack-type (ST) chicken sausages spontaneously fermented (no starter) and with the addition of the bioprotective (sakacin K)-producing *Latilactobacillus sakei* CTC494.

			Log_10_ reduction* during corrective storage	
Product		Temperature (°C)	After ripening	After HPP
FT	No starter	4	NI**	0.18 ± 0.08
			NI	0.17 ± 0.16
	+CTC494	4	NI	0.25 ± 0.05
		15	NI	0.29 ± 0.06
ST	No starter	4	1.04 ± 0.61	0.37 ± 1.21
		15	0.85 ± 0.16	0.81 ± 0.63
	+CTC494	4	NI	0.53 ± 0.82
		15	NI	0.93 ± 0.36

*Mean ± standard deviation. **No inactivation.

As a general rule for FT sausages, *L. monocytogenes* was not affected by the application of post-processing strategies. No inactivation was observed during 7 days of corrective storage (irrespective of the temperature, 4 or 15°C). The application of HPP after ripening or after the corrective storage did not result in a relevant inactivation of the pathogen. In the case of ST sausages, the corrective storage did not significantly decrease the levels of *L. monocytogenes*, while HPP inactivated the pathogen to a variable extent without statistically significant differences (*p* > 0.05) between the application of HPP after ripening or after the corrective storage. Despite the variability, the average Log_10_ reduction due to HPP in ST without bioprotective culture was significantly higher (*p* < 0.05) than in ST sausages with *L. sakei* CTC494.

The limited effect of the corrective storage in both FT and ST sausages could be related to the short time and/or the low temperature. Under growth-preventing conditions of DFS, the higher the temperature (closer to the optimal growth) the faster the non-thermal inactivation of vegetative pathogens due to metabolic exhaustion mechanisms (Serra-Castelló et al., 2021). For instance, 1 Log_10_ reduction of *L. monocytogenes* was achieved after 6–9 days of storage at 25°C in dry-cured ham with *a*_*w*_ 0.85–0.87, but considerably longer times were needed at 15°C (Serra-Castelló et al., 2020). Also, Porto-Fett et al. (2022) observed, in *fuet* (with *a*_*w*_ 0.89 and 0.86), no significant reduction of *L. monocytogenes* during their storage at 20°C for 7 days. However, the pathogen counts were reduced as the storage time increased up to 30 days, being greater for *fuet* with the lowest *a*_*w*_ value (0.86).

The difference in the listericidal efficacy of HPP in FT and ST sausages could be attributed to the low *a*_*w*_ values of FT sausages (0.827–0.853) compared to those of ST sausage, which is known to exert a piezo-protective effect on bacteria against HPP (Rubio et al., 2013; Bover-Cid et al., 2015; Balamurugan et al., 2020). In agreement with this fact, Rubio et al. (2018) demonstrated a 2.2 Log_10_ reduction of *L. monocytogenes* after an HPP treatment of 475 MPa/6.25 min in DFS with high *a*_*w*_ (0.92) compared with non-detectable reductions when the same treatment was applied to products with low *a*_*w*_ (<0.86), which is consistent with similar results previously obtained with dry-cured ham of different *a*_*w*_ (Bover-Cid et al., 2015). Moreover, the pathogen resistance against processing and preservation technologies is determined by several factors, including the physiological status of the bacterial cells. The gradual acidification and drying occurring during sausage fermentation and ripening have been recognized to trigger stress responses before the harsh conditions become lethal, thus conferring cross-protection among different factors and reducing the inactivation (Ross et al., 2008). This has important consequences. On the one hand, the validation studies through challenge tests cannot be performed by inoculating the final product but the meat batter to expose the pathogens to the whole process. Regarding HPP, alternative strategies to increase the efficacy have been suggested, such as applying the technological treatment at earlier stages of the process. Marcos et al. (2005) assessed the application of HPP on sausages just stuffed before the fermentation, however, the results were not satisfactory because it disrupted the proper fermentation. Another study states that the pathogen HPP inactivation (*E. coli* O157:H7) in DFS is most effective when HPP treatment is applied before the meat matrix reaches an *a*_*w*_ of 0.90 (Balamurugan et al., 2020).

Taking this into consideration, in the present study, HPP was also applied to ST sausages just after the fermentation, i.e., once the LAB reached the maximum population density to take advantage that the pH reached the minimum value, while showing a high *a*_*w*_ (0.965). The results are shown in Figure 3 (dotted lines). The inactivation of *L. monocytogenes* was 4.1 ± 0.7 Log_10_ for ST without starter and 2.77 ± 1.34 Log_10_ units for ST with bioprotective starter, though the difference was not statistically significant due to the wide variability among the three sampled sausages. During the subsequent ripening, the levels of the pathogen remained stable. Noteworthy, the concentration of *L. monocytogenes* in these ST sausages was not significantly different (*p* > 0.05) from the ST sausages pressurized at the end of the ripening. Therefore, taking into account the complexity of the logistics associated with an eventual application of HPP to unfinished sausages (e.g., before the end of the ripening), our results suggest that this strategy is not worth nor feasible from the industrial perspective.

## Conclusion

The present study allows identifying the role of the different processing conditions, corrective storage, and high-pressure processing for the control of *L. monocytogenes* in chicken low-acid dry-fermented sausages. Temperature is the most critical factor during the first stages and the low *a*_*w*_ at the end of the ripening. The action of the bioprotective bacteriocin-producing starter culture (*L. sakei* CTC494) prevented the growth of *L. monocytogenes* in the snack-type sausages and enhanced the inactivation in *fuet*-type sausages, which could be related to the higher lactic acid production and consequent decrease of pH, but also to the production of the antilisterial bacteriocin sakacin k. Corrective storage strategy at 4 and 15°C for up to 7 days does not appear as an effective strategy to enhance the reduction of the pathogen in the final product. HPP treatment is suitable to promote *L. monocytogenes* reduction as long as the *a*_*w*_ is not too low to avoid piezo-protection. Our results provide guidance to the food industry to design effective strategies (e.g., fermentation temperature, bioprotective starter cultures, HPP) to control *L. monocytogenes* in chicken dry-fermented sausages.

## Data availability statement

The datasets presented in this article are not readily available because restricted. Requests to access the datasets should be directed to corresponding author.

## Author contributions

SB-C, AJ, and PG contributed to conception and design of the study. AA-C, AJ, and CS-C contributed experimental work and organized the database. AA-C, CS-C, PG, and SB-C performed and interpreted the statistical analysis and simulations. AA-C wrote the first draft of the manuscript. CS-C and SB-C wrote sections of the manuscript. All authors contributed to manuscript revision, read, and approved the submitted version.
